# Toward personalized medicine for mal de débarquement syndrome

**DOI:** 10.3389/fneur.2026.1824869

**Published:** 2026-06-22

**Authors:** Jun Maruta, Catherine Cho, Sergei B. Yakushin

**Affiliations:** 1 Department of Neurology, Icahn School of Medicine at Mount Sinai, New York, NY, United States; 2 Department of Rehabilitation and Human Performance, Icahn School of Medicine at Mount Sinai, New York, NY, United States; 3 Department of Neurology, NYU Grossman School of Medicine, New York, NY, United States; 4 Department of Otolaryngology, NYU Grossman School of Medicine, New York, NY, United States

**Keywords:** gravity, heterogeneity, multidisciplinary management, precision medicine, quality of life, questionnaire, vestibular habituation

## Abstract

**Background:**

Mal de débarquement syndrome (MdDS) is a chronic vestibular disorder of non-spinning vertigo, with various somatic, cognitive, and affective symptoms. The manifestation of MdDS is often only subjective, and different symptoms are variably emphasized when patients appraise the severity of their illness. The etiology of MdDS remains unclear but may lie in improperly sustained neuroplasticity in the velocity storage mechanism of the central vestibular system. Two broad approaches targeting velocity storage—one focusing on correcting velocity storage’s maladapted behavior and the other on attenuating its contribution to higher-order processing—have yielded varying degrees of treatment success.

**Methods:**

We conducted a secondary analysis of data from our recent study contrasting the outcomes of the above two approaches. We examined the variations in individual emphases of separate symptoms by correlating the subjective ratings of overall MdDS severity with those of specific symptoms across time points for each subject. We also explored the possibility that variations in symptom presentation influence the responsiveness to the two approaches of treatment.

**Results:**

Many symptoms correlated with the overall severity rating, but even those that showed strong correlation among many subjects, such as dizziness, fatigue, and brain fog, were endorsed variably across subjects. A moderate unfavorable dependence on visual sensitivity was found for the responsiveness to the velocity storage attenuation treatment, which distinguished the two treatment approaches.

**Conclusion:**

Results provide a glimpse into the complexity of MdDS manifestations. Individual variations in what contributes to their perceived overall symptom severity may aid the choice of treatment approach.

## Introduction

Mal de débarquement syndrome (MdDS) is a chronic vestibular disorder associated with a continuous false sensation of self-motion that is often familiarly experienced after exposure to passive motion, such as a boat or plane ride, except that, for patients with this condition, the disorienting sensation lingers perpetually (1–3). The primary symptom is collectively described as non-spinning vertigo, consisting of any combination of sensations of oscillatory motion, such as rocking, swaying, or bobbing, and gravitational pull, as though being pulled in a particular direction (1–5).

The neurological etiology and factors that contribute to the development of MdDS are still not clear. While a long-held consensus has been that MdDS stems from neural plasticity rather than damage, i.e., a failure to readapt to a stable environment after adapting to being in motion, further understanding of the illness remained elusive for a long time (1, 2, 6). However, the insight by the clinical scientist, Mingjia Dai, that a central vestibular mechanism known as velocity storage may be maladapted in MdDS opened opportunities to directly address the root cause of the illness (5, 7, 8).

Velocity storage was originally characterized as a common drive that underlies a prolonged nystagmic response or after-response to rotational vestibular or optokinetic stimulation, expressing a phenomenon of “central inertia” or “storage,” and its working has been most extensively approached through objective quantification in terms of visual-vestibular interactions (9–12). Although velocity storage is thus closely associated with the vestibulo-ocular reflex (VOR), it can be activated, and nystagmus generated, by other cues of sustained rotation, such as when a person follows a rotating wall surround with an extended arm or when stepping around on a rotating floor (13, 14). Further, velocity storage is dynamically maintained to act as a “neural gyroscope” so that nystagmus is reoriented when there is a change in the gravito-inertial reference (15–18). An important link to MdDS is that velocity storage is malleable in its ability to sustain or reorient the output and, thus, may be maladapted (8, 19). Also importantly, velocity storage is thought to contribute to the sense of orientation in space and the perception of self-motion (11–13, 20, 21).

Although evidence for the velocity storage involvement in the pathophysiology of MdDS has thus far been indirect, we have reported considerable success with two broad categories of treatment approaches that by design target velocity storage, one focusing on correcting its spatial orientation properties as pioneered by Dai (“*VOR readaptation*”) and the other on generally attenuating its contribution, if maladapted, to higher-order processing (“*velocity storage attenuation*”) (5, 22–25). The success of Dai’s treatment approach in particular has been independently replicated by others (26–31).

Despite such progress, many patients do not benefit from available treatments, let alone medication but also velocity storage-based approaches (3, 22, 23, 32). Part of the problem may stem from the multifacetedness of MdDS symptomatology and disconnection from objective means to gauge the severity of symptoms that are only subjectively experienced by patients (23). Namely, the internal sensation of self-motion or imbalance is often not expressed as physical signs in MdDS (5, 23). Further, the symptoms of non-spinning vertigo are typically accompanied by those of somatic complaints (e.g., fatigue, vestibular migraine, headache, and visually induced dizziness), reduced cognitive function (e.g., decreased attention and short-term memory), and affective problems (e.g., depression and anxiety); these symptoms negatively impact patients’ quality of life and their ability to work and contribute to the society (4, 22, 33, 34). Critically, the evaluation of treatment efficacy depends on assessment of, and monitoring changes in, the overall severity of MdDS as self-reported by patients (5, 22–25), but such assessment is further complicated by individual differences in the emphasis of various symptoms attributed to the illness (23, 35).

Here, we firstly sought to shed more light on the variations in individual emphases of separate symptoms while appraising the overall severity of MdDS. Secondly, we explored the possibility that the responsiveness to treatments could depend on such variations. Our recent report contrasted the effects of the *VOR readaptation* and *velocity storage attenuation* regimens in the treatment of patients with MdDS (23). We thus conducted a secondary analysis of data from that study.

## Methods

### Sample

The methods of patient recruitment and treatment interventions are detailed in the previously published primary report (23). Pertinently to the present study, applicants seeking treatment were screened with an intake form, and each candidate’s diagnosis of MdDS (chronic continuous non-oscillatory vertigo that improves with passive motion) with an associable motion trigger was confirmed by a board-certified physician, through a telephone interview when necessary. Data from a total of 43 patient subjects with MdDS (mean (SD) age: 47.1 (14.0) years old; 83.7% women) who underwent their respectively assigned treatment regimens, typically over the course of 5 days, were analyzed. Assignment to the two intervention groups was random. Twenty subjects underwent the VOR readaptation regimen, whereas the remaining 23 subjects underwent the velocity storage attenuation regimen. All interventions were provided by the last author. The VOR readaptation regimen involved a smooth periodic head tilt maneuver about the roll or pitch axis during a full-field, unidirectional optokinetic stimulus (OKS) about an earth-vertical axis generated in a cylindrical enclosure. The combined stimulus introduced to the subject a visual motion element that oscillated about an axis orthogonal to both the OKS and the head tilt, i.e., pitch when the head was rolled or roll when pitched. This “fictitious” motion in the non-inertial frame of reference was applied to counter the phantom sensation of rocking or swaying (5, 8). On the other hand, the velocity storage attenuation regimen was designed to achieve its purpose by introducing conflict between two velocity storage-mediated responses, namely optokinetic nystagmus and the VOR during sinusoidal side-to-side rotation about an earth-vertical axis at a frequency at which the velocity storage mechanism dominates the VOR response (36). The study protocol was reviewed and approved by the Institutional Review Board of Icahn School of Medicine at Mount Sinai (protocol number 19–00877, initial approval August 25, 2020).

### Symptom questionnaires

Subjects were asked to self-evaluate their symptoms in two separate forms during the intake process, on each day of the laboratory visit, and at 2-week, and 1-, 3-, and 6-month follow-ups. First, the overall severity of MdDS condition, considering not only the sensation of self-motion but also all other symptoms attributable to MdDS, was subjectively reported on a single 11-point scale of 0–10, where the score 0 indicated no symptoms and 10 the most difficult of combined symptoms that the subject could imagine. Second, subjective intensities of ten specific symptoms of possible relevance to MdDS, namely, brain fog, sensitivity to computer screen, ear fullness, fatigue, sensitivity to fluorescent lights, fuzzy vision, head pressure, headache, tinnitus, and dizziness (22, 37), were reported on a symptom subscale questionnaire, also on an 11-point scale.

In addition to the symptom questionnaires, subjects were asked to complete the Dizziness Handicap Inventory (DHI) during the intake process and at 2-week, and 1-, 3-, and 6-month follow-ups. The DHI is an easy-to-complete 25-item questionnaire that aims to quantify perceived disability and handicap caused by dizziness (38–40). The questionnaire has a total added score ranging from 0 (no handicap) to 100 (significant handicap) and is divided into physical, emotional, and functional subscales. We note that despite the wide use and the seeming relevance of the questionnaire items to the context of dizziness symptoms, the validity of the DHI has been questioned (39, 40). Moreover, the DHI is dissociated from day-to-day changes in symptoms as questionnaire items are evaluated in terms of the patient’s experience over some period of time. Here, as the DHI’s presumed construct is only indirectly relevant to the objectives of the present study, we present its outcomes simply as a reference probe for the overall MdDS symptom severity measure.

### Statistical analysis

**Figure 1:**
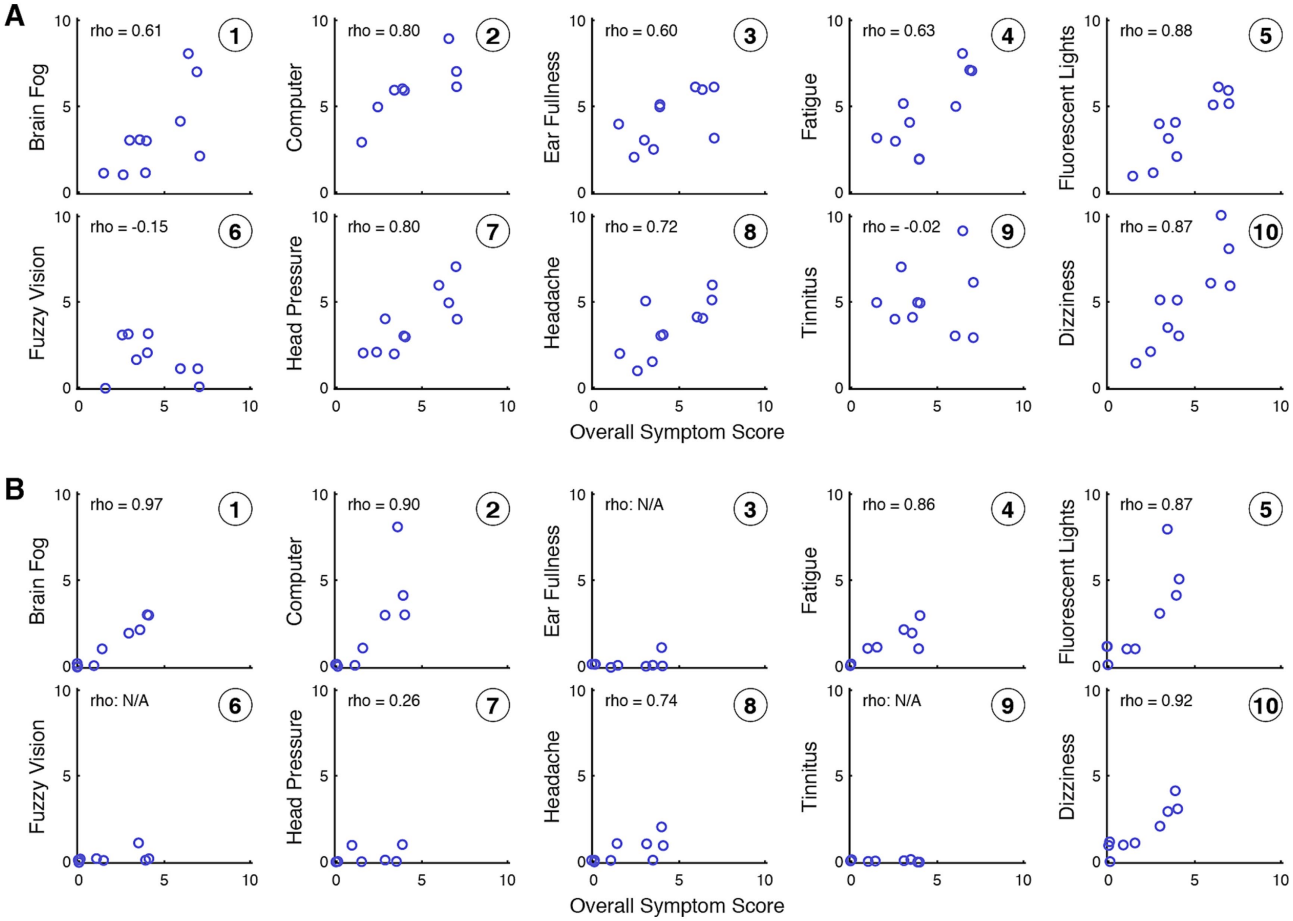
Examples showing individually different patterns of relationships between one’s perceived overall severity of MdDS and various specific symptoms. Small random noise was added so that overlapping responses can be distinguished in the plot. **(A)** Correlograms of a 58-year-old woman whose MdDS was attributed to a cruise (Subject 24). She reported a substantial reduction in the overall severity of her MdDS over the five-day course of the velocity storage attenuation regimen but subsequently experienced a mild relapse. **(B)** Correlograms of a 49-year-old woman whose MdDS was attributed to a 7-h car ride (Subject 37). She reported having almost no symptoms after the first day of undergoing VOR readaptation and throughout the remaining course of her treatment, but within 2 weeks, she experienced a substantial relapse. “N/A” signifies that the rho-value could not be calculated because of a general lack of symptom endorsement.

Data analysis was aided by scripts written with MATLAB (The MathWorks, Natick, MA, USA). We probed the validity of the overall MdDS severity rating by computing Spearman correlation coefficients (rho) against DHI scores. Possible discrepancies in the strengths of correlation were inspected using Fisher’s z-transformation. To examine the variations in individual emphases of separate symptoms, we computed rho-values between the ratings of overall severity of MdDS across time points and contemporaneous ratings of specific symptoms for each subject (Figure 1). Each subject provided a range of scores during the period of observation, due to factors such as the laboratory intervention, subjects’ engaged activities, hormonal changes, or spontaneous fluctuations, generally permitting such computations (23). However, when a subject never, or rarely and negligibly endorsed a specific symptom in the subscale questionnaire across time points, with an arbitrary cut-point of mean rating < 0.2 on the 0–10 scale, a value of zero was assigned to rho to signify the symptom’s negligible contribution to the subject’s perception of MdDS overall severity. The strength of correlation was interpreted according to a guide suggested for behavioral sciences, such that 0 ≤ |rho| < 0.2 is interpreted as negligible, 0.2 ≤ |rho| < 0.4 as weak, 0.4 ≤ |rho| < 0.6 as moderate, 0.6 ≤ |rho| < 0.8 as strong, and 0.8 ≤ |rho| ≤ 1 as very strong (41). Then for each symptom, the across-individual distribution of rho-values was examined in terms of deviation of their mean (*μ*) from zero using a t-test. The alpha level was set at 0.05, corrected for multiple comparison using Holm’s method (42). The effect size d, defined as the mean rho-value divided by the corrected sample standard deviation, was also examined and interpreted conforming to behavioral sciences guidelines (43).

To explore a possible dependence of treatment responsiveness on a specific symptom that presented before the treatment intervention, we first operationalized responsiveness by normalizing the change in a given subject’s overall severity rating between the first and last days of treatment as a ratio of the latter relative to the former (22, 23, 25). In this scheme, the value 1 indicated no change in the severity rating while the value 0 indicated a symptom-free condition on the last day. Then for each intervention group, we computed rho-values between the normalized changes and pre-treatment ratings of each specific symptom from the subscale questionnaire, and tested against the null hypothesis that there was no monotonic association between the two. Adjustments for multiple comparison across symptoms were not made in this examination because of its exploratory nature. Between-group differences in rho-values were examined with an application of Fisher’s z-transformation.

## Results

### Variations in individual emphases of separate symptoms

Subjects’ appraisals of the overall severity of their MdDS condition were generally reflected on their perceived handicaps. The correlation between the DHI total and overall symptom severity scores varied between weak (rho = 0.37, *p* = 0.015) and strong (rho = 0.65, *p* < 0.001) depending on the timing of assessment, but these extremes were not statistically significantly different (*p* = 0.081); therefore, the effect of timing was not further considered. The overall symptom severity rating was similarly generally positively correlated with the DHI subscales as well, broadly supporting its ability to gauge how one perceives the impact of the illness.

Subjects’ perceived intensities of various specific symptoms were related to individual appraisals of overall MdDS severity in diverse manners. Figure 1 shows correlograms representing such relations for two example subjects. For the first subject (Subject 24), the perceived intensities of sensitivity to fluorescent lights and dizziness were most closely, and very strongly, correlated with her appraisal of the overall severity of her condition with a rho-value at 0.88 and 0.87, respectively (Figure 1A, ⑤ and ⑩). Also very strongly correlated with her overall severity appraisal were sensitivity to computer screens and head pressure (② and ⑦). On the other hand, while she experienced tinnitus and fuzzy vision, the intensities of these symptoms were not associated with the overall severity of her MdDS (⑨ and ⑥). The remaining parameters from the symptom subscale questionnaire, namely, brain fog, ear fullness, fatigue, and headache, showed moderate to strong correlation to her overall severity appraisal. For the second subject (Subject 37), the perceived intensities of brain fog and dizziness were most closely, and very strongly, correlated with her appraisal of the overall MdDS severity with a rho-value at 0.97 and 0.92, respectively (Figure 1B, ① and ⑩). Sensitivities to computer screens and fluorescent lights as well as fatigue were also very strongly correlated with the overall severity of her MdDS (②, ⑤, and ④). Headache also showed a strong correlation (⑧). In contrast, ear fullness, fuzzy vision, head pressure, or tinnitus were not significant features of her MdDS experience.

**Figure 2:**
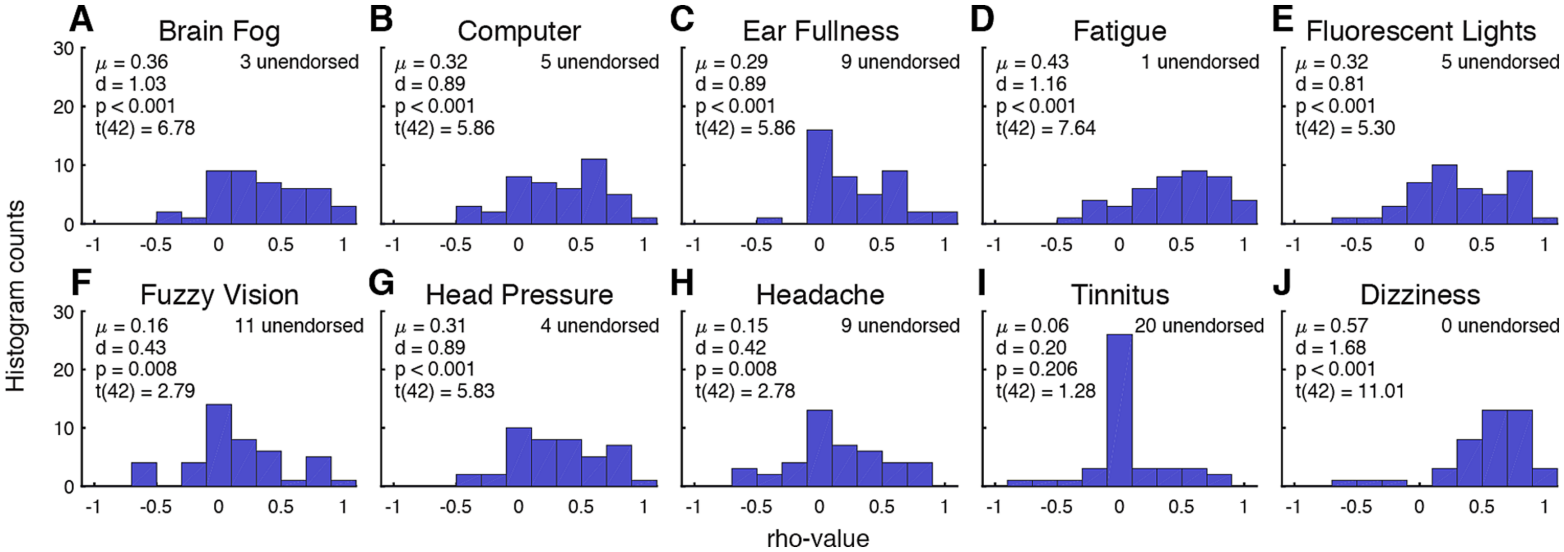
Across-subject variations in the correlation between the intensity of various specific symptoms and the contemporaneous perception of the overall severity of MdDS. **(A)**-**(J)** Histograms of correlation values for symptom subscale items. When a particular symptom was not reported as part of a subject’s experience, rho was set to zero to signify the negligible contribution of the symptom to this subject’s perception of MdDS severity. The number of such incidents is shown in an inset within each panel.

Across subjects, dizziness was the parameter that was most commonly and most strongly correlated with individual appraisals of the overall severity of their MdDS condition (Figure 2J). Because MdDS is primarily characterized by a continuous false sensation of self-motion, the common presence of this association (*p* < 0.001) with a very large effect size (d = 1.68) was expected. However, individual rho-values varied widely, and their mean was just at a moderate level of 0.57, indicating that factors other than dizziness contributed to the shaping of many individuals’ overall severity appraisals. In 5 subjects (12%), the perceived intensity of dizziness was negligibly or even inversely correlated with their overall severity appraisals. In three of these cases (Subjects 10, 19, and 43), different parameters from our symptom subscale questionnaire were found with moderate to strong correlations to their respective overall severity appraisals, possibly yielding a greater influence than dizziness on their perception. By contrast, in one case (Subject 2), the term “dizziness” unfortunately appears to have failed to describe the motion sensations that this individual qualitatively described as “rocking, swaying, and bobbing,” contributing to the particular dissociation. Also unfortunately, in this and the remaining case (Subject 1), factors that may have shaped these individuals’ appraisals of the overall MdDS severity could not be identified among the parameters specified in our symptom subscale questionnaire, and thus remain unknown.

Fatigue, brain fog, ear fullness, sensitivities to computer screens and fluorescent lights, and head pressure were other parameters that were commonly associated with the overall severity appraisal across subjects (*p* < 0.001) and yielded a large effect size (d > 0.8) (Figures 2A–E,G). Nevertheless, similarly to dizziness, the rho-values for the correlation between these parameters and the overall appraisal varied widely across subjects. The mean rho-values generally fell within the range of a weak strength, except for fatigue, which registered as moderate. Fuzzy vision and headache also demonstrated common presence of association with the overall severity appraisal (*p* = 0.008), but with a small effect size (d < 0.5) and more typically at a negligible level or often with a negative correlation (Figures 2F,H). Lastly, and rather uniquely among the subscale questionnaire items, the intensity of tinnitus did not commonly correlate with the overall appraisal of MdDS severity (*p* = 0.21) (Figure 2I). Indeed, nearly half of the subjects did not identify tinnitus as part of their MdDS experience, while some subjects strongly or even very strongly associated it with their MdDS severity.

### The relation between baseline symptoms and treatment responsiveness

**Figure 3:**
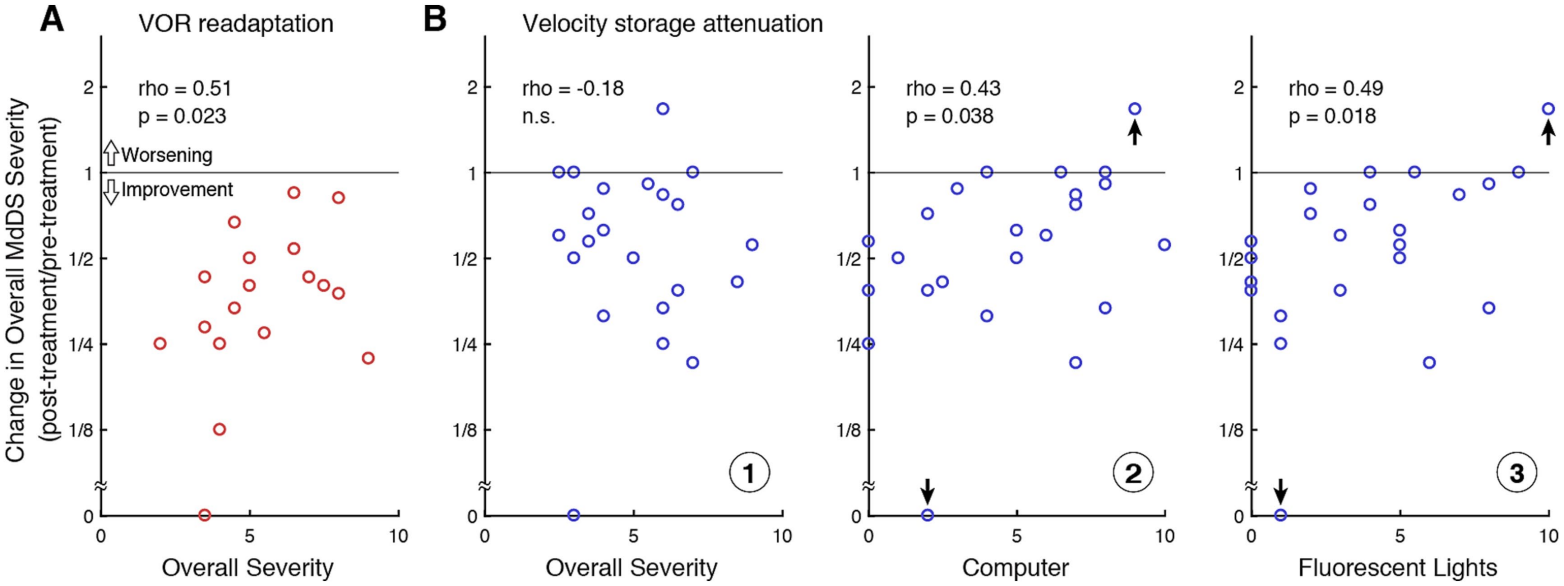
Relationship between pre-treatment symptom rating and treatment responsiveness. Each symbol represents a subject. Symbols below the horizontal line indicate those with a lower overall severity score reported on the last day than on the first day. Also see Table 1. **(A)** VOR readaptation, overall severity. **(B)** Velocity storage attenuation. ① Overall severity. n.s.: not significant. ② Sensitivity to computer screens. ③ Sensitivity to fluorescent lights. Special care was additionally taken to examine the possibility of significant bias that may have been contributed by the points indicated by black arrows.

A possible dependence of treatment responsiveness on the subjects’ perceived pre-treatment symptom intensity was explored using Spearman correlation (Table 1). For the VOR readaptation group, there was not a statistically significant dependence on any of the specific symptoms identified in the subscale questionnaire. However, those with a more severe perception of their overall MdDS condition at baseline tended to be less responsive to the treatment (rho = 0.51) (Figure 3A). Despite having overall lower efficacy, such dependence was not found for the velocity storage attenuation group (rho = −0.18) (Figure 3B, ①), and the group difference was statistically significant (*p* = 0.024, Table 1). For the velocity storage attenuation group, on the other hand, there was a statistically significant and moderate dependence on sensitivity to computer screens (rho = 0.43) and sensitivity to fluorescent lights (rho = 0.49) (Figure 3B, ② and ③). The correlation for the latter was also statistically different from the corresponding correlation in the VOR readaptation group (*p* = 0.045, Table 1).

**Table 1:** Spearman correlations between the responsiveness to an intervention and symptoms presented before the intervention.

Scale	VOR readaptation		Velocity storage attenuation		Group difference
	(*n* = 20)		(*n* = 23)		
	rho	p	rho	p	*p*
Overall severity	**0.51**	**0.023**	−0.18	0.40	**0.024**
Brain fog	−0.10	0.67	0.35	0.10	0.16
Computer	0.18	0.44	**0.43**	**0.038**	0.40
Ear fullness	−0.02	0.93	0.25	0.25	0.40
Fatigue	−0.02	0.93	0.30	0.17	0.32
Fluorescent lights	−0.13	0.59	**0.49**	**0.018**	**0.045**
Fuzzy vision	−0.03	0.89	0.30	0.16	0.29
Head pressure	0.28	0.23	0.06	0.80	0.48
Headache	−0.13	0.58	0.12	0.59	0.45
Tinnitus	−0.19	0.43	−0.05	0.82	0.67
Dizziness	0.32	0.17	0.23	0.29	0.78

Bold typeface indicates *p* < 0.05. A positive rho-value indicates a tendency for those with a high baseline symptom rating to have reported less reduction in their MdDS severity following the treatment compared to their peers. Also see Figure 3.

Effects of possible outliers were additionally investigated. One subject (Subject 15) in the velocity storage attenuation group reported transient overall worsening of her MdDS condition during the course of the treatment regimen, citing an increase in visual sensitivity. As stated previously (23), we attributed this turn of events to the visual-vestibular conflict that was progressively intensified per protocol. Since this subject also indicated high sensitivities to computer screens and fluorescent lights before the treatment, her data may have falsely skewed the results toward a larger dependence of treatment responsiveness on these parameters (Figure 3B, ② and ③, upward black arrow); therefore, the correlations for the group were re-computed without this subject’s data. The statistical significance in the correlations between treatment responsiveness and pre-treatment intensities of these two symptoms was lost, but the strength of correlation for sensitivity to computer screens was still close to moderate (rho = 0.37, *p* = 0.093), and that for sensitivity to fluorescent lights remained moderate (rho = 0.41, *p* = 0.055). The latter correlation was still statistically larger than the corresponding correlation in the VOR readaptation group (*p* = 0.044, one-tailed).

On the opposite side of the scale, another subject in the group (Subject 16) became free of symptoms after the treatment, and this subject initially reported low sensitivities to computer screens and fluorescent lights (Figure 3, ② and ③, downward black arrow). Once again, to rule out the possibility of false skewing of results, correlations for the group were re-computed without this subject’s data. The correlation for sensitivity to computer screens lost the statistical significance but was still close to moderate (rho = 0.39, *p* = 0.069), while the correlation for sensitivity to fluorescent lights remained both statistically significant and moderate (rho = 0.45, *p* = 0.034), as well as statistically significantly larger than the corresponding correlation in the VOR readaptation group (*p* = 0.033, one-tailed). Without either Subject 15 or Subject 16, the correlation between pre-treatment sensitivity to fluorescent lights and treatment responsiveness was still close to moderate although statistically non-significant (rho = 0.37, *p* = 0.099).

These results support that the responsiveness to treatment can depend on the symptom profile of an individual patient. Specifically, the response to VOR readaptation is moderately dependent on the overall severity rating, and the response to velocity storage attenuation is moderately dependent on visual sensitivity.

## Discussion

Our correlation analysis indicated that many discrete symptoms contributed to the shaping of individual patients’ perception of their MdDS severity, in keeping with the multifacetedness of MdDS. Critically, however, even those symptoms that showed a strong correlation to the overall severity rating among many individuals, such as dizziness, fatigue, and brain fog, were endorsed variably across individuals. On the other hand, while tinnitus was least commonly experienced among our sample cohort, it was nevertheless strongly or very strongly expressed with MdDS in some individuals. This finding supports the idea that dizziness with tinnitus often suggests peripheral problems, but not always so, including but not limited to MdDS and vestibular migraine (37, 44–47). Our results confirm that how patients appraise the severity of their MdDS condition is deeply personal with varied emphases on different symptoms.

Standardized questionnaires, such as based on scaling of common symptoms, may be useful in identifying and comparing across individuals the presence and severity of some problems, such as anxiety (48, 49), depression (50), fatigue (51, 52), or motion sickness susceptibility (53, 54). With MdDS in contrast, the varied emphases placed on different symptoms that individual patients attribute to their condition suggest a fallacy in any attempt to compare the severity of the illness among patients. Results support that to guide clinical decisions, there is instead a need for a better way to characterize their individual experience of MdDS. Certainly, traditional treatment approaches to MdDS target the management of specific symptoms. For example, while MdDS is not a psychiatric disorder per se, it is often accompanied by psychiatric symptoms as are many vestibular disorders (55–58). Benzodiazepines, selective serotonin reuptake inhibitors, and serotonin-norepinephrine reuptake inhibitors that are often used for treating anxiety, stress, and depression, have been found to provide relief in some patients (32, 45, 59–61). Similarly, patients with MdDS with co-existing vestibular migraine reportedly respond well to migraine prophylaxis (32). It is also said that alleviating psychological distress, especially stigma associated with the rare and underrecognized status and subjective nature of MdDS, may improve quality of life of patients (35).

However, the argument for individual-centric medicine for MdDS is not to advocate focusing on isolated symptoms that may have come about secondarily to underlying primary causes. The recent revelation that the velocity storage mechanism may be involved in MdDS opened opportunities for positive long-term outcomes by targeting the root cause of the illness under a unified vestibular system-based perspective (5, 22–25). Thus-derived approaches have yielded broad clinical success. Still, the unfortunate fact that those including VOR readaptation and velocity storage attenuation have been ineffective in many patients does need explanation (5, 22–24, 31, 62). To this extent, our present exploratory examination of the relation between baseline symptoms and treatment responsiveness suggests that variations in symptom presentation may influence how patients respond to these treatments. The veracity of and bases for such influence must be scrutinized. For example, one reason why a high overall perception of the severity of MdDS, as opposed to any particular symptom we surveyed, may reduce patients’ responsiveness to VOR readaptation could be because of individually varying comorbidities whose symptoms are attributed to MdDS but instead need to be treated separately or in parallel. Similarly, if visual sensitivity confounds the intended effect of the visual-vestibular conflict introduced in the velocity storage attenuation treatment, clinical judgement should be made to patients’ benefit for shorter training sessions and/or more gradual progression, or a different approach.

In discussing individual-centric medicine, the terms precision medicine and personalized medicine are often used interchangeably. However, some draw a distinction in that precision medicine characterizes and approaches diseases with their intrinsic biology, often in terms of molecular parameters, while personalized medicine emphasizes treating the patient as a unique individual, rather than as a member of a group, by additionally considering the individual’s environmental and psychosocial background (63–66). A key foundation of precision-medicine is taxonomy/stratification/subtyping of diseases. Therefore, its approach to predict, diagnose, treat, or prevent human disease is by nature reductionistic, but may still be a necessary step toward personalized medicine (63–66).

As MdDS is regarded as an illness of neuroplasticity, i.e., acquired through experience, framing the malleability of the velocity storage mechanism at the center of MdDS etiology and treatment may be where our approach first deviates from a more restricted sense of precision medicine toward personalized medicine. Nevertheless, lessons from the precision medicine approach are valuable. Taxonomy in precision medicine is established through common biological bases rather than clinical symptoms, such that previously separately identified diseases that share a common biological cause may be connected while disease subtypes with distinct biological causes be distinguished (65). Notably, precision medicine is advanced iteratively through pilot studies with expanding scope and scale, supported by knowledge of the mechanisms of fundamental biological processes (65, 67). Disorders that may be associated with abnormal functioning of velocity storage include vestibular migraine, high motion sickness susceptibility, and vestibular syncope, which in turn are, like MdDS, portrayed with heterogeneity and overlapping symptomatology (32, 36, 47, 68–74). Accordingly, henceforward these and possibly other disorders may need to be placed within the scope of MdDS awareness in clinical or research settings and be characterized with common parameters, both subjective and objective, covering symptom, demographic, velocity storage, and other domains. These parameters need to be established initially through small scale exploratory studies, and then sufficient data must be accumulated to permit multidimensional analysis. Equally important is basic and theoretical research that drive mechanistic explanation of MdDS (8, 62, 75–80).

## Conclusion

The current study, as inherent to secondary research, is limited by its exploratory nature and dependence on data already collected not specific for the purpose. Also, inherent to MdDS symptomatology, the utilized measures were entirely subjective in nature. Nevertheless, our results provide a glimpse into the complexity of MdDS manifestations—how patients appraise the severity of their MdDS condition is deeply personal with varied emphases on different symptoms. However, the possibility that maladaptation of the velocity storage mechanism underlies MdDS presents a unified vestibular system-based perspective, and may facilitate precision and personalized medicine approaches to this illness. Choosing the treatment approach based on individual patients’ symptom profiles is an important step in this direction.

## Data Availability

The original contributions presented in the study are included in the article/Supplementary material, further inquiries can be directed to the corresponding authors.
