# Supplementary material for: Toward personalized medicine for mal de débarquement syndrome
Source: Front Neurol. 2026 Jun 22;17:1824869. doi: 10.3389/fneur.2026.1824869 (PMC13333527; doi:10.3389/fneur.2026.1824869)
Supplement: Supplementary file 1 [file Data_Sheet_1.PDF]

Demographics

VOR readaptation

ID	Age (yrs)	Sex (0=F,1=M)
1	39	0
4	57	0
8	62	0
11	37	1
13	27	0
18	45	0
21	35	0
22	61	0
23	36	0
25	34	0
26	55	0
27	77	0
28	39	0
29	34	0
30	78	1
33	35	1
37	49	0
38	55	1
41	41	0
47	38	1

Velocity storage attenuation

ID	Age (yrs)	Sex (0=F,1=M)
2	52	0
3	22	0
6	55	0
7	74	0
9	47	0
10	25	1
12	66	0
14	32	0
15	32	0
16	58	0
17	49	0
19	50	0
20	61	0
24	58	0
31	41	0
32	44	0
34	44	0
35	36	0
36	67	0
39	34	1
42	52	0
43	62	0
46	30	0

Overall symptoms

VOR readaptation

ID	Intake	Day1	Day2	Day3	Day4	Day5	2wk	1mo	3mo	6mo
1	5	7	4.5	5	2	3	3	3.5	3.5	3.5
4	4	4.5	4	3	3.5	1.5	1.5	1.5	3.5	4
8	6.5	6.5	6.5	3.5	6.5	3.5	6.5	6.5	6.5	6.5
11	3	4	2	2	1.5	0.5	1.5	1.25	2	2
13	6	6.5	7	6.5	6.5	5.5	6.5	4.5	0	0
18	6	2	2	2	1	0.5	3	2	3	1
21	7	3.5	4.5	3	4	0	4	4	3	2
22	6	4	2	3	2	1	4	4	2	0
23	6.5	5	5	4.5	4	2	3	3	2	3
25	5	5.5	4.5	1.5	1.5	1.5	5.5	4	5.5	6
26	10	8	3	7	6	6.5	5	5	9	8
27	10	3.5	2	8	0	0	8	3	3	2
28	5	3.5	3	1.5	1.5	1.5	1.5	1.5	1.5	1.5
29	5	3.5	3.5	3	2	1	1.5	1.5	2	1.5
30	5	4.5	4.5	4	3.5	3	4.5	3.5	4.5	3.5
33	7.5	7.5	3.5	2.75	2.75	3	3	3	2.5	2.5
37	5	3.5	0	1	0	0	3	4	1.5	4
38	8	8	3	3	2	3	2	3	6	8
41	5	5	2.5	3.5	5	2.5	2.5	2.5	2.5	2.5
47	8	9	6	6	6	2	6	6	8	4

Velocity storage attenuation

ID	Intake	Day1	Day2	Day3	Day4	Day5	2wk	1mo	3mo	6mo
2	9.5	8.5	8.5	8.5	2.5	3.5	1.5	0.5	3.5	2
3	6	5	5	4	4	2.5	3.5	3.5	4	5
6	9	6	5	3.5	6	1.5	1	1.5	0.5	0.5
7	6	2.5	4	5	3	2.5	2.5	6	4	1
9	6.5	6.5	6	5	5	5	5	5	4.5	5
10	7	3.5	1	1	1	2.5	2.5	3.5	3.5	2.5
12	3.5	6.5	5.5	4.5	4.5	2.5	1.25	1.75	1	0.5
14	8	6	6	6	5	5	4	1	0	0
15	6	6	6	6	6	10	6	5	N/A	2
16	6	3	3	2	0	0	1	0.25	0	1
17	6	2.5	6	5.5	2	1.5	2.5	2	1.5	2.5
19	9	9	6.5	6.5	6.5	5	7	7	7	6
20	5	4	4	1	1	1.25	2.25	2.25	2.5	2
24	6.5	7	6	7	3	1.5	4	2.5	4	3.5
31	4	4	3	3	3	2.5	2.5	2.5	3.5	3.5
32	3.5	3.5	3	3	2	2	3	2	2	2
34	7	7	7	8	6	7	7	9	7	7
35	7	3	3	2	3	3	3	4	2	3
36	8	6.5	7	3	2.5	2.5	0.5	1.6	1.5	4.5
39	6	6	4.5	3	2	2	2	8	5	2
42	4	3	3	1.5	0.75	1.5	1	1.5	1.5	1.5
43	6.5	5.5	5.5	5.5	3.5	5	6.5	6.5	5.5	7
46	7	4	3.5	4	3.5	3.5	5.5	2.75	2.5	2

Brain fog

VOR readaptation

ID	Intake	Day1	Day2	Day3	Day4	Day5	2wk	1mo	3mo	6mo
1	N/A	4.5	3	5	5	5	4	0	3	6.5
4	N/A	5	4	1	0	0	0	0	1	1
8	N/A	4	3	2	2	2	3	3	0	3
11	N/A	4.5	4.5	3.5	3.5	1.5	2	2	2.5	1
13	N/A	2	1	1	0	0	0	0	3	0
18	N/A	2	2	1	0	0	2	0	1	1
21	N/A	3	2	3	2	2	N/A	7	N/A	3
22	N/A	2	0	4	2	1	1	3	3	0
23	N/A	4	N/A	2	2	2	2	4	1	2
25	N/A	5	3	3	2	3	1	1	4	3
26	2	1	0	2	0	0	2	3	2	2
27	5	4	3	3	10	N/A	2	0	3	0
28	2	1	0	0	0	0	0	0	0	N/A
29	N/A	3	4	6	4	N/A	6	1	2	1
30	N/A	7	7	7	7	7	6	5	5	2.5
33	7	3	1	3	1	2	2	2	2	0
37	N/A	2	0	0	0	0	2	3	1	3
38	9	5	5	4	6	6	9	5	9	9
41	3.5	3	2	2	2	4	4	3.5	3	3.5
47	7	6	6	6	6	5	5	6	N/A	3

Velocity storage attenuation

ID	Intake	Day1	Day2	Day3	Day4	Day5	2wk	1mo	3mo	6mo
2	N/A	0	1	4	4	0	3.5	3	6	7
3	N/A	5	1.5	3.5	2.5	2.5	3	N/A	2	5
6	N/A	6	5	4	6	4	1	1	1	1
7	N/A	7	5	6	6	6	4	4	3	2
9	7	5	6	4	5	5	5	4	3	2
10	N/A	2	1	0	0	1	0	2	7	2
12	N/A	1	1	0	1	0	0	0	0	0
14	N/A	6	6	5	5	5	4	2	N/A	0
15	N/A	9	6	4	4	3	4	4	N/A	2
16	N/A	0	0	0	0	0	0	0	0	0
17	N/A	3	3	0	0	0	2	2	1	3
19	4	3	4	4	3	2	3	3	3	2
20	N/A	0	0	0	0	0	0	0	0	0
24	8	7	4	2	3	1	1	1	3	3
31	0	0	0	2	0	0	0	2	3	2
32	5	1	1	1	1	1	3	2	1.5	3
34	0	3	1	2	1	0	2	N/A	2	2
35	3	1	0	0	0	0	4	1	2	2
36	N/A	1	0	0	0	0	0	0	0	0
39	N/A	5	5	3	4	2	6	5	2.5	0.5
42	N/A	0	1	1	0	0	N/A	0	1	0
43	4	7	N/A	4	1	N/A	N/A	N/A	4	N/A
46	7	3	4	2	3	2	N/A	2	2	2.5

Sensitivity to computer screen

VOR readaptation

ID	Intake	Day1	Day2	Day3	Day4	Day5	2wk	1mo	3mo	6mo
1	N/A	9	9	9	9	9	9	8.5	8	9
4	N/A	6	2	1	1	1	2	1	1	0
8	N/A	0	0	0	0	0	0	0	0	0
11	N/A	3.5	2.5	1.5	3.5	1.5	1.5	0.5	1.5	0.5
13	N/A	7	6	6	4	4	3	2	0	0
18	N/A	4	4	1	1	1	2	1	1	1
21	N/A	3	2	1	2	2	N/A	1	N/A	2
22	N/A	8	5	7	5	5	3	4	4	0
23	N/A	4	N/A	3	2	1	5	5	1	2
25	N/A	4	4	2	2	2	0	0	5	8
26	9	9	6	4	3	5	7	5	8	8
27	0	0	0	0	0	N/A	0	0	1	1
28	0	0	0	0	0	0	1	0	0	N/A
29	N/A	3	2	3	5	N/A	N/A	1	0	0
30	N/A	6	7	7	5	5	5	4	4.5	2
33	3	8	6	7.5	7	7	5	6	3	2
37	N/A	8	0	0	0	0	3	3	1	4
38	4	3	1	2	2	2	7	3	7	7
41	3.5	3	4	4	4	5	5	5	4	2.5
47	5	5	6	6	5	5	4	5	N/A	2

Velocity storage attenuation

ID	Intake	Day1	Day2	Day3	Day4	Day5	2wk	1mo	3mo	6mo
2	N/A	2.5	0	4	4	0	3	3.5	7	7
3	N/A	5	5.5	5	4.5	6.5	4	N/A	4.5	8
6	N/A	0	0	0	0	0	0	0	0	0
7	N/A	8	7	5	5	5	5	7	5	7
9	N/A	7	6	5	6	6	4	4	4	6
10	N/A	2	2	2	2	2	3	4	5	6
12	N/A	0	0	0	0	0	0	0	0	0
14	N/A	7	8	6	5	5	2	1	N/A	0
15	N/A	9	8	9	10	10	8	6	N/A	2
16	N/A	2	2	1	0	1	1	0	1	0
17	N/A	6	0	0	0	0	3	5	0	2
19	10	10	9	10	9	9	8	7	5	7
20	N/A	4	3	2.5	3	2.5	3.5	2.5	3	2
24	9	7	N/A	6	N/A	3	6	5	6	6
31	8	5	1	2	2	3	2	3	5	5
32	7	0	0	0	0	0	0	1	1	1
34	6	6.5	4	5	5	4	7	N/A	2	5
35	10	4	0	2	2	1	4	4	6	3
36	N/A	2	0	2	2	1	1.5	0	2	4
39	N/A	8	5	4	2	1	7.5	6.5	5	4
42	N/A	1	0	0	0	0	N/A	0	0	0
43	9	8	N/A	8	7	N/A	N/A	N/A	6	N/A
46	8	3	5	3	3	3	N/A	3	2	3

Ear fullness

VOR readaptation

ID	Intake	Day1	Day2	Day3	Day4	Day5	2wk	1mo	3mo	6mo
1	N/A	0	0	0	0	0	0	0	3	0
4	N/A	1	0	0	0	0	0	0	0	1
8	N/A	0	0	0	0	0	0	0	0	0
11	N/A	2.5	3.5	1.5	0.5	0.5	0	0	0.5	0
13	N/A	0	0	0	0	0	0	0	0	0
18	N/A	0	3	1	2	2	3	1	1	0
21	N/A	1	1	2	2	2	N/A	4	N/A	0
22	N/A	4	0	3	1	1	0	2	1	0
23	N/A	3	N/A	3	3	3	4	2	1	2
25	N/A	0	0	1	1	1	1	0	1	1
26	1	3	0	1	0	1	3	3	8	5
27	0	3	1	1	0	N/A	0	0	2	0
28	0	0	0	0	0	0	0	0	0	N/A
29	N/A	3	2	3	2	N/A	1	1	1	3
30	N/A	3	1	1	1	0	1	1	0.5	0
33	3	1	1	1	0	0	0	2	0	0
37	N/A	0	0	0	0	0	0	0	0	1
38	2	3	3	1	2	1	4	3	4	4
41	3.5	2	3	2	2	1	2	2	3	2.5
47	2	0	0	2	2	0	0	0	N/A	0

Velocity storage attenuation

ID	Intake	Day1	Day2	Day3	Day4	Day5	2wk	1mo	3mo	6mo
2	N/A	0	0	4	5	1	2	1	7	9
3	N/A	4	3	2	2.5	3.5	2	N/A	1.5	2
6	N/A	0	0	0	0	0	0	0	0	0
7	N/A	5	3	3	2	2	2	6	4	4
9	2	2	2	0	2	2	1	1	0	0
10	N/A	3	5	2	2	2	2	2	5	3
12	N/A	3	2	1	2	0	0	0	0	0
14	N/A	6	5	6	4	4	3	1	N/A	0
15	N/A	4	3	3	3	3	3	3	N/A	2
16	N/A	3	4	2	0	0	1	1	1	1
17	N/A	4	3	0	1	1	0	1	1	4
19	8	2	6	3	2	2	3	1	1	2
20	N/A	0	0	0	0	0	0	0	0	0
24	6	6	6	3	3	4	5	2	5	2.5
31	0	0	0	0	0	0	0	0	0	0
32	1	2	1	1	1	0	2	2	1.5	2
34	0	2	2	3	1	3	1.5	N/A	5	2
35	2	1	0	0	0	0	2	1	0	2
36	N/A	0	0	0	0	0	0	0	0	0
39	N/A	8	5	4	3	3	3.5	3.5	3.5	2.5
42	N/A	0	0	0	0	0	N/A	0	0	0
43	N/A	8	N/A	1	0	N/A	N/A	N/A	5	N/A
46	3	7	6	3	7	7	N/A	3	4	5

Fatigue

VOR readaptation

ID	Intake	Day1	Day2	Day3	Day4	Day5	2wk	1mo	3mo	6mo
1	N/A	7	6	5	6	6	7	5	8	8.5
4	N/A	6	2	1	1	1	0	0	2	4
8	N/A	4	2	2	3	2	3	3	4	4
11	N/A	0.5	7.5	0.5	0.5	0.5	2	0	0	2
13	N/A	4	5	5	4	4	2	2	2	1
18	N/A	2	1	1	1	0	1	0	2	1
21	N/A	7	5	5	7	4	N/A	6	N/A	5
22	N/A	3	0	5	2	2	1	1	3	0
23	N/A	3	N/A	4	2	2	4	5	2	5
25	N/A	6	5	3	4	4	2	1	2	2
26	2	1	0	3	0	3	3	2	3	2
27	10	6	3	6	10	N/A	8	2	8	3
28	5	3	1	0	1	0	3	2	4	N/A
29	N/A	3	2	2	1	N/A	1	1	1	0
30	N/A	7	3	4	3	4	5	5	5.5	2
33	6	2	2	1	0	2	3	2	1	1
37	N/A	2	0	1	0	0	2	1	1	3
38	10	5	5	3	3	5	7	7	7	7
41	3.5	5	4	4	3	5	5	4	3	4.5
47	8	8	5	6	5	3	5	6	N/A	4

Velocity storage attenuation

ID	Intake	Day1	Day2	Day3	Day4	Day5	2wk	1mo	3mo	6mo
2	N/A	2.5	4	0	4	0	4	3	5	7
3	N/A	0	0	3.5	0	2.5	2	N/A	2.5	3.5
6	N/A	7	5	3	3	5	2	1	1	1
7	N/A	7	6	4	5	5	7	7	7	4
9	7	4	4	5	7	5	4	4	3	4
10	N/A	5	4	2	3	4	2	2	5	2
12	N/A	2	1	1	1	1	0	0	0	0
14	N/A	6	6	6	5	5	5	2	N/A	0
15	N/A	9	7	8	8	10	8	7	N/A	3
16	N/A	4	3	2	0	1	1	0	1	0
17	N/A	1	0	1	0	0	1	2	2	3
19	4	6	3	6	2	2	5	5	4	5
20	N/A	0	0	0	0	0	1	0.5	0	0
24	8	7	5	7	5	3	2	3	2	4
31	0	0	0	5	1	1	0	1	4	1
32	5	3	1	1	1	1	2	2	0.5	3
34	2	3	4	3	1	1	4	N/A	8	3
35	7	4	2	2	3	1	8	9	3	3
36	N/A	2	2	1	0	0	0	0	1	0
39	N/A	5	5	4	4	3	8.5	8.5	4.5	2.5
42	N/A	3	1	1	1	1	N/A	2	2	1
43	8	8	N/A	1	1	N/A	N/A	N/A	6	N/A
46	9	6	7	7	6	5	N/A	4	3	3

Fluorescent lights

VOR readaptation

ID	Intake	Day1	Day2	Day3	Day4	Day5	2wk	1mo	3mo	6mo
1	N/A	9	8	9	9	9	10	8.5	10	10
4	N/A	2	2	2	1	1	1	0	1	1
8	N/A	7	5	3	3	0	3	0	0	2
11	N/A	1.5	0.5	0.5	0.5	0.5	0	0	0	0
13	N/A	1	0	0	0	0	1	0	0	0
18	N/A	0	0	0	0	0	0	0	1	0
21	N/A	3	2	1	4	3	N/A	3	N/A	2
22	N/A	8	5	7	5	5	4	4	4	0
23	N/A	7	N/A	4	3	3	5	5	2	5
25	N/A	5	4	2	2	3	1	0	5	6
26	N/A	5	2	3	3	5	2	3	2	2
27	N/A	4	3	3	3	N/A	4	1	3	5
28	0	0	0	0	0	0	1	0	0	N/A
29	N/A	0	2	2	4	N/A	N/A	1	0	0
30	N/A	2	1	0	0	1	1	1	0.5	0
33	3	2	2	0	0	0	1	2	0	0
37	N/A	8	0	1	1	1	3	4	1	5
38	9	3	2	3	2	3	9	5	9	9
41	3.5	3	5	5	5	5.5	5	5	4	3.5
47	7	7	6	5	6	5	5	5	N/A	2

Velocity storage attenuation

ID	Intake	Day1	Day2	Day3	Day4	Day5	2wk	1mo	3mo	6mo
2	N/A	0	0	0	0	0	7	6.5	10	8
3	N/A	5	5.5	5	4.5	6.5	4	N/A	4	5.5
6	N/A	1	0	0	0	0	0	0	0	0
7	N/A	9	6	6	4	4	7	7	7	8
9	4	4	5	3	3	3	2	2	2	2
10	N/A	2	2	2	2	2	3	5	5	5
12	N/A	0	0	0	0	0	0	0	0	0
14	N/A	7	7	6	5	5	3	1	N/A	1
15	N/A	10	10	10	10	10	8	8	N/A	4
16	N/A	1	2	1	1	0	1	1	0	1
17	N/A	3	0	0	0	0	0	0	0	0
19	6	5	5	5	5	5	4	3	2	0
20	N/A	1	1	1	1	0.5	1	0	0	0
24	6	6	5	5	4	1	2	1	4	3
31	8	5	1	2	1	1	1	2	5	4
32	4	0	0	0	0	0	0	0	1	2
34	6	5.5	5	6	5	5	8	N/A	7	5
35	10	4	1	2	2	2	4	4	7	4
36	N/A	3	3	2	2	1	0	2	2	7
39	N/A	8	6	5	4	4	6.5	6.5	6.5	6
42	N/A	0	0	0	0	0	N/A	0	0	0
43	9	8	N/A	8	10	N/A	N/A	N/A	8	N/A
46	4	2	3	3	2	1	N/A	3	3	1

Fuzzy vision

VOR readaptation

ID	Intake	Day1	Day2	Day3	Day4	Day5	2wk	1mo	3mo	6mo
1	N/A	4	2	1	1	0	3	7.5	8	2.5
4	N/A	6	5	1	2	1	1	1	1	1
8	N/A	8	0	0	0	0	0	0	0	0
11	N/A	0.5	0.5	0.5	0.5	0.5	0	0	0	0
13	N/A	0	0	0	0	0	0	0	0	0
18	N/A	5	4	3	1	2	3	1	2	1
21	N/A	0	0	0	0	0	N/A	0	N/A	0
22	N/A	4	0	4	2	1	3	3	0	0
23	N/A	2	N/A	0	0	0	0	0	0	2
25	N/A	0	0	0	0	0	0	0	0	1
26	2	0	0	0	0	0	0	0	1	2
27	5	4	3	4	0	N/A	3	0	4	3
28	0	0	0	0	0	0	0	0	0	N/A
29	N/A	0	2	0	1	N/A	0	0	0	0
30	N/A	1	0	1	1	0	1	1	0.5	0
33	2	0	0	0	0	0	0	0	0	0
37	N/A	1	0	0	0	0	0	0	0	0
38	5	4	3	2	3	3	8	3	8	8
41	6	3	3	3	3.5	5	4	4.5	5	4.5
47	6	0	0	0	0	0	0	0	N/A	0

Velocity storage attenuation

ID	Intake	Day1	Day2	Day3	Day4	Day5	2wk	1mo	3mo	6mo
2	N/A	0	0	0	0	0	0	0	4	1
3	N/A	0	0	1.5	1.5	4.5	3	N/A	3.5	3.5
6	N/A	0	0	0	0	0	0	0	0	0
7	N/A	0	1	0	1	1	1	1	1	0
9	4	2	4	2	3	2	2	3	3	2
10	N/A	0	0	0	0	0	0	2	4	0
12	N/A	0	0	0	1	1	1	0	0	0
14	N/A	1	4	2	1	1	0	0	N/A	0
15	N/A	7	3	3	4	5	3	3	N/A	2
16	N/A	1	0	0	0	0	0	0	0	0
17	N/A	0	0	0	0	0	0	3	1	0
19	1	2	1	2	2	2	1	1	1	2
20	N/A	0	0	0	0	0	0	0	0	0
24	10	0	1	1	3	0	2	3	3	1.5
31	1	0	0	0	0	0	0	1	0	0
32	4	0	0	0	0	0	0	1	0.5	0.5
34	1	0	0	0	0	0	1	N/A	1	0
35	10	5	0	1	1	0	4	4	5	4
36	N/A	0	0	0	0	0	0	0	0	0
39	N/A	3	2	2	1	0	1.5	0	1	1.5
42	N/A	0	0	0	0	0	N/A	0	0	0
43	0	0	N/A	0	0	N/A	N/A	N/A	0	N/A
46	3	3	3	3	3	3	N/A	3	3	4

Head pressure

VOR readaptation

ID	Intake	Day1	Day2	Day3	Day4	Day5	2wk	1mo	3mo	6mo
1	N/A	7	4	7	7	7	4	3	5	5.5
4	N/A	1	0	0	0	0	0	0	0	1
8	N/A	0	0	0	0	0	0	0	0	0
11	N/A	7.5	5.5	2.5	2.5	1.5	1	1	2.5	1.5
13	N/A	9	9	9	9	9	7	5	0	0
18	N/A	2	2	3	1	2	3	1	2	1
21	N/A	6	1	4	7	4	N/A	7	N/A	0
22	N/A	3	0	5	3	1	2	3	2	0
23	N/A	2	N/A	4	3	3	2	1	1	1
25	N/A	2	1	1	1	1	0	0	0	0
26	4	7	0	5	1	3	3	5	5	3
27	10	3	3	3	2	N/A	0	0	8	0
28	0	1	0	0	0	0	0	0	0	N/A
29	N/A	5	2	2	1	N/A	1	3	4	3
30	N/A	7	6	7	3	7	6	5	6	2.5
33	6	5	2	3	1	1	1	3	1	0
37	N/A	0	0	1	0	0	0	0	0	1
38	9	5	4	6	7	7	9	6	9	9
41	3.5	5	3	2	2	3	4	3	3	3.5
47	5	2	5	5	5	5	2	2	N/A	2

Velocity storage attenuation

ID	Intake	Day1	Day2	Day3	Day4	Day5	2wk	1mo	3mo	6mo
2	N/A	2.5	3	5	5	2	3	1	7	8
3	N/A	4	3	3.5	2.5	4.5	4	N/A	2.5	3.5
6	N/A	0	0	0	0	0	0	0	0	0
7	N/A	3	4	4	2	2	4	3	2	2
9	4	2	3	2	3	3	2	3	3	1
10	N/A	0	1	0	2	0	0	2	5	2
12	N/A	3	1	0	1	0	0	0	0	0
14	N/A	4	5	6	4	5	4	2	N/A	0
15	N/A	4	3	3	5	5	4	4	N/A	2
16	N/A	3	4	2	0	0	1	1	0	0
17	N/A	2	2	1	1	1	3	5	5	4
19	9	2	6	5	2	2	3	2	2	4
20	N/A	1	0	0	1	0	2	1.5	1	0.5
24	5	7	6	4	4	2	3	2	3	2
31	5	0	1	0	1	0	0	5	3	5
32	5	2	2	1	1	0	3	2	1.5	3
34	0	2	1	2	2	0	2	N/A	3	5
35	2	1	1	0	1	0	3	1	1	1
36	N/A	0	0	0	0	0	0	0	0	0
39	N/A	7	7	5	3	3	5	3.5	1.5	1.5
42	N/A	1	0	0	1	0	N/A	1	0	1
43	1	4	N/A	0	0	N/A	N/A	N/A	4	N/A
46	7	5	4	7	5	5	N/A	3	3	3

Headache

VOR readaptation

ID	Intake	Day1	Day2	Day3	Day4	Day5	2wk	1mo	3mo	6mo
1	N/A	5	4	7	6	5	6	6.5	9	5.5
4	N/A	0	0	0	0	0	0	0	0	1
8	N/A	0	0	0	0	0	0	1	1	0
11	N/A	5.5	5.5	1.5	0.5	0.5	1	0.5	1.5	1
13	N/A	3	0	0	0	0	0	0	0	0
18	N/A	3	2	3	2	2	4	2	2	2
21	N/A	7	2	4	7	5	N/A	6	N/A	5
22	N/A	0	0	0	0	0	0	0	1	0
23	N/A	2	N/A	4	3	2	3	4	1	2
25	N/A	0	0	0	0	0	0	0	0	1
26	4	2	0	4	0	0	1	3	5	5
27	10	4	4	4	2	N/A	2	0	7	5
28	1	1	0	0	0	0	1	0	0	N/A
29	N/A	0	0	1	0	N/A	1	0	0	0
30	N/A	3	1	3	1	0	1	1	1	1
33	5	0	0	0	1	0	0	1	1	0
37	N/A	0	0	0	0	0	1	1	1	2
38	9	5	5	5	7	7	8	8	8	8
41	3.5	5	3	0	2	1	2	3.5	4	4.5
47	5	5	7	6	5	5	5	5	N/A	3

Velocity storage attenuation

ID	Intake	Day1	Day2	Day3	Day4	Day5	2wk	1mo	3mo	6mo
2	N/A	0	4	5	3	2	4	3.5	9	9
3	N/A	0	0	1.5	0.5	4.5	2	N/A	3.5	5.5
6	N/A	0	0	0	0	0	0	0	0	0
7	N/A	3	1	0	1	1	3	2	2	3
9	N/A	0	0	1	1	0	3	3	2	3
10	N/A	0	0	0	5	0	2	3	4	2
12	N/A	0	0	0	0	0	0	0	0	0
14	N/A	1	1	3	2	5	1	0	N/A	0
15	N/A	4	4	5	6	6	8	6	N/A	2
16	N/A	0	0	0	0	0	0	0	0	1
17	N/A	1	1	1	1	1	0	5	5	3
19	3	3	1	3	2	2	1	1	1	0
20	N/A	0	0	0	0	0	0	0	1	0
24	4	6	4	5	5	2	3	1	3	1.5
31	5	0	N/A	0	1	0	0	5	1	5
32	4	0	3	2	0	0	2	2	0.5	0.5
34	5	0	0	1	1	0	0.5	N/A	3	2
35	1	0	1	1	4	0	5	2	4	1
36	N/A	0	1	0	0	0	0	0	0	0
39	N/A	6	7	2	2	2	6.5	5	3	2
42	N/A	0	0	1	1	0	N/A	0	0	1
43	0	0	N/A	0	0	N/A	N/A	N/A	0	N/A
46	4	3	4	6	3	2	N/A	2	3	3

Tinnitus

VOR readaptation

ID	Intake	Day1	Day2	Day3	Day4	Day5	2wk	1mo	3mo	6mo
1	N/A	0	0	0	0	0	0	0	0	0
4	N/A	5	1	1	0	1	1	1	5	1
8	N/A	0	0	0	0	0	0	0	0	0
11	N/A	0.5	0.5	0.5	0.5	0.5	0	0	1	0
13	N/A	0	0	0	0	0	0	0	0	0
18	N/A	0	0	0	0	0	0	0	0	0
21	N/A	5	4	6	7	5	N/A	6	N/A	6
22	N/A	0	0	0	0	0	0	0	1	0
23	N/A	6	N/A	2	2	1	5	3	2	4
25	N/A	0	0	0	0	0	0	0	0	0
26	3	3	1	3	1	1	3	3	8	8
27	10	7	6	4	0	N/A	4	2	9	3
28	0	0	0	0	0	0	0	0	0	N/A
29	N/A	0	1	1	3	N/A	1	1	1	0
30	N/A	0	0	0	0	0	0	0	0	0
33	0	0	0	0	0	0	0	0	0	0
37	N/A	0	0	0	0	0	0	0	0	0
38	4	0	0	0	0	0	0	1	0	0
41	3.5	1	1	1	2	0.5	3	2.5	4	4.5
47	0	0	0	0	0	0	0	0	N/A	0

Velocity storage attenuation

ID	Intake	Day1	Day2	Day3	Day4	Day5	2wk	1mo	3mo	6mo
2	N/A	0	0	0	0	0	0	2	10	3
3	N/A	0	0.5	0	1.5	2.5	2	N/A	1	1.5
6	N/A	2	0	0	0	0	0	0	1	0
7	N/A	2	2	1	1	1	1	0	0	0
9	N/A	0	0	0	0	0	0	0	0	0
10	N/A	0	0	0	0	0	0	0	0	0
12	N/A	5	3	3	3	3	3	3	3	3
14	N/A	1	1	3	2	1	0	0	N/A	0
15	N/A	4	3	3	2	2	3	3	N/A	2
16	N/A	0	0	0	0	0	0	0	0	0
17	N/A	0	0	0	0	0	0	0	0	0
19	0	2	1	1	1	1	1	1	1	0
20	N/A	0	0	0	0	0	1	0.5	0	0
24	9	6	3	3	7	5	5	4	5	4
31	0	0	0	0	0	0	0	0	0	0
32	1	0	0	0	0	0	0	0	0	0.5
34	0	0	0	0	0	0	0	N/A	0	0
35	4	0	0	0	0	0	1	1	0	0
36	N/A	0	0	0	0	0	0	2	5	2.5
39	N/A	6	5	3	2	1	1.5	0	1.5	1
42	N/A	0	0	0	0	0	N/A	0	0	0
43	10	10	N/A	10	10	N/A	N/A	N/A	9	N/A
46	0	0	0	0	0	0	N/A	0	1	2

Dizziness

VOR readaptation

ID	Intake	Day1	Day2	Day3	Day4	Day5	2wk	1mo	3mo	6mo
1	N/A	6	5	5	6	5	8	7.5	8	6.5
4	N/A	4	4	3	3	4	2	1	3	4
8	N/A	7	7	5	5.5	5	6.5	7	7	7
11	N/A	4.5	3.5	2.5	1.5	1.5	0.5	0	1.5	1
13	N/A	9	9	9	9	9	6	6	0	1
18	N/A	2	3	3	1	1	4	2	3	2
21	N/A	6	5	4	4	4	N/A	4	N/A	2
22	N/A	4	0	6	4	2.5	2	4	3	0
23	N/A	5	N/A	5	4	4	5	5	1	2
25	N/A	7	4	1	1	2	1	0	4	5
26	10	7	3	3	2	7	5	5	6	7
27	10	6	4	8	8	N/A	10	2	8	4
28	3	3	3	2	2	2	2	2	1	N/A
29	N/A	6	4	2	2	N/A	3	3	2	3
30	N/A	7	6	6	4	4	6	5	5.5	3.5
33	5	4	3	3	2	3	3	4	3	3
37	N/A	3	0	1	1	1	2	3	1	4
38	9	3	3	2	1	2	8	3	8	8
41	3.5	5	2.5	3.5	4	5	3	3	3	4.5
47	9	10	8	8	8	7	7	7	N/A	4

Velocity storage attenuation

ID	Intake	Day1	Day2	Day3	Day4	Day5	2wk	1mo	3mo	6mo
2	N/A	0	0	0	0	0	0	1	0	5
3	N/A	5	5.5	6	4	5.5	4	N/A	4.5	7.5
6	N/A	7	5	4	7	9	1	3	1	1
7	N/A	8	5	7	4	4	5	7	6	3
9	N/A	5	6	5	5	6	5	5	4	5
10	N/A	6	7	3.5	3	6	2	5	4	3
12	N/A	6	4.5	3	4	3	1	2	1	1
14	N/A	4	1	3	2	2	0	0	N/A	0
15	N/A	8	7	8	9	10	7	6	N/A	2
16	N/A	1	2	1	0	0	0	0	0	0
17	N/A	3	2	3	2	2	2	1	2	2.5
19	9	6	6	8	6	5	5	4	3	5
20	N/A	3	2	1.5	2	1	2.5	2.5	2.5	2
24	10	8	6	6	5	1.5	3	2	5	3.5
31	8	4	3	4	3	3	2	2	3	4
32	10	3	3	3	2	2	3	2	1.5	2.5
34	7	7	7	8	7	7	7	N/A	9	7
35	9	3	0	1	3	1	4	2	2	3
36	N/A	5	5	3	2	2	0	2	3	4.5
39	N/A	6	5	4	3	3	6.5	5.5	3.5	1.5
42	N/A	4	3	2	2	1	N/A	1	1	1
43	6	9	N/A	6	6.5	N/A	N/A	N/A	7	N/A
46	7	4	6	3	4	3	N/A	3	3	3.5

Dizziness Handicap Inventory, Physical

VOR readaptation

ID	Intake	Day1	Day2	Day3	Day4	Day5	2wk	1mo	3mo	6mo
1	20	N/A	N/A	N/A	N/A	N/A	20	16	14	14
4	18	N/A	N/A	N/A	N/A	N/A	8	2	14	18
8	12	N/A	N/A	N/A	N/A	N/A	12	14	14	14
11	6	N/A	N/A	N/A	N/A	N/A	4	4	4	4
13	2	N/A	N/A	N/A	N/A	N/A	6	0	4	0
18	8	N/A	N/A	N/A	N/A	N/A	2	2	4	4
21	16	N/A	N/A	N/A	N/A	N/A	N/A	16	16	8
22	12	N/A	N/A	N/A	N/A	N/A	12	16	18	0
23	16	N/A	N/A	N/A	N/A	N/A	14	12	8	10
25	14	N/A	N/A	N/A	N/A	N/A	2	2	8	8
26	4	N/A	N/A	N/A	N/A	N/A	8	6	16	12
27	8	N/A	N/A	N/A	N/A	N/A	12	4	2	4
28	10	N/A	N/A	N/A	N/A	N/A	12	4	6	4
29	6	N/A	N/A	N/A	N/A	N/A	4	2	0	2
30	10	N/A	N/A	N/A	N/A	N/A	10	10	8	8
33	2	N/A	N/A	N/A	N/A	N/A	14	10	10	8
37	10	N/A	N/A	N/A	N/A	N/A	6	20	4	10
38	20	N/A	N/A	N/A	N/A	N/A	16	14	16	16
41	10	N/A	N/A	N/A	N/A	N/A	12	6	10	10
47	2	N/A	N/A	N/A	N/A	N/A	2	4	4	4

Velocity storage attenuation

ID	Intake	Day1	Day2	Day3	Day4	Day5	2wk	1mo	3mo	6mo
2	12	N/A	N/A	N/A	N/A	N/A	8	6	N/A	10
3	20	N/A	N/A	N/A	N/A	N/A	22	12	18	18
6	4	N/A	N/A	N/A	N/A	N/A	2	0	2	0
7	18	N/A	N/A	N/A	N/A	N/A	18	18	14	14
9	12	N/A	N/A	N/A	N/A	N/A	12	16	16	18
10	14	N/A	N/A	N/A	N/A	N/A	12	18	14	12
12	6	N/A	N/A	N/A	N/A	N/A	4	4	4	4
14	16	N/A	N/A	N/A	N/A	N/A	12	2	N/A	0
15	12	N/A	N/A	N/A	N/A	N/A	12	12	N/A	10
16	16	N/A	N/A	N/A	N/A	N/A	14	6	2	6
17	6	N/A	N/A	N/A	N/A	N/A	0	2	0	0
19	18	N/A	N/A	N/A	N/A	N/A	10	6	4	2
20	8	N/A	N/A	N/A	N/A	N/A	8	4	8	2
24	16	N/A	N/A	N/A	N/A	N/A	10	6	8	6
31	2	N/A	N/A	N/A	N/A	N/A	6	4	4	4
32	14	N/A	N/A	N/A	N/A	N/A	8	14	10	10
34	16	N/A	N/A	N/A	N/A	N/A	12	6	12	16
35	24	N/A	N/A	N/A	N/A	N/A	22	18	14	20
36	22	N/A	N/A	N/A	N/A	N/A	10	18	18	18
39	12	N/A	N/A	N/A	N/A	N/A	10	10	6	8
42	0	N/A	N/A	N/A	N/A	N/A	0	0	0	N/A
43	16	N/A	N/A	N/A	N/A	N/A	14	16	16	16
46	22	N/A	N/A	N/A	N/A	N/A	N/A	10	10	12

Dizziness Handicap Inventory, Emotional

VOR readaptation

ID	Intake	Day1	Day2	Day3	Day4	Day5	2wk	1mo	3mo	6mo
1	25	N/A	N/A	N/A	N/A	N/A	24	22	20	20
4	12	N/A	N/A	N/A	N/A	N/A	4	2	10	14
8	14	N/A	N/A	N/A	N/A	N/A	14	14	16	16
11	14	N/A	N/A	N/A	N/A	N/A	4	6	4	2
13	12	N/A	N/A	N/A	N/A	N/A	9	8	2	0
18	6	N/A	N/A	N/A	N/A	N/A	6	4	4	4
21	26	N/A	N/A	N/A	N/A	N/A	N/A	28	32	10
22	12	N/A	N/A	N/A	N/A	N/A	4	8	8	0
23	14	N/A	N/A	N/A	N/A	N/A	12	14	6	12
25	14	N/A	N/A	N/A	N/A	N/A	4	4	8	8
26	10	N/A	N/A	N/A	N/A	N/A	8	8	14	10
27	10	N/A	N/A	N/A	N/A	N/A	10	2	4	2
28	22	N/A	N/A	N/A	N/A	N/A	10	2	8	12
29	16	N/A	N/A	N/A	N/A	N/A	6	8	4	6
30	24	N/A	N/A	N/A	N/A	N/A	20	12	16	12
33	16	N/A	N/A	N/A	N/A	N/A	10	12	10	6
37	26	N/A	N/A	N/A	N/A	N/A	20	18	18	18
38	24	N/A	N/A	N/A	N/A	N/A	26	20	26	26
41	12	N/A	N/A	N/A	N/A	N/A	10	9	16	8
47	10	N/A	N/A	N/A	N/A	N/A	10	10	10	10

Velocity storage attenuation

ID	Intake	Day1	Day2	Day3	Day4	Day5	2wk	1mo	3mo	6mo
2	16	N/A	N/A	N/A	N/A	N/A	12	6	N/A	14
3	16	N/A	N/A	N/A	N/A	N/A	4	8	8	2
6	24	N/A	N/A	N/A	N/A	N/A	6	4	4	0
7	6	N/A	N/A	N/A	N/A	N/A	8	12	10	12
9	22	N/A	N/A	N/A	N/A	N/A	16	16	12	14
10	24	N/A	N/A	N/A	N/A	N/A	12	10	12	10
12	4	N/A	N/A	N/A	N/A	N/A	4	2	4	6
14	20	N/A	N/A	N/A	N/A	N/A	24	4	N/A	0
15	22	N/A	N/A	N/A	N/A	N/A	18	18	N/A	16
16	6	N/A	N/A	N/A	N/A	N/A	2	0	0	2
17	20	N/A	N/A	N/A	N/A	N/A	8	8	16	14
19	16	N/A	N/A	N/A	N/A	N/A	18	16	14	12
20	18	N/A	N/A	N/A	N/A	N/A	12	12	10	6
24	24	N/A	N/A	N/A	N/A	N/A	6	6	8	20
31	12	N/A	N/A	N/A	N/A	N/A	6	8	10	8
32	6	N/A	N/A	N/A	N/A	N/A	4	6	4	4
34	12	N/A	N/A	N/A	N/A	N/A	8	9	12	8
35	28	N/A	N/A	N/A	N/A	N/A	26	28	18	20
36	20	N/A	N/A	N/A	N/A	N/A	10	14	12	14
39	14	N/A	N/A	N/A	N/A	N/A	16	16	6	4
42	8	N/A	N/A	N/A	N/A	N/A	0	2	2	N/A
43	16	N/A	N/A	N/A	N/A	N/A	12	10	8	14
46	24	N/A	N/A	N/A	N/A	N/A	N/A	14	16	14

Dizziness Handicap Inventory, Functional

VOR readaptation

ID	Intake	Day1	Day2	Day3	Day4	Day5	2wk	1mo	3mo	6mo
1	31	N/A	N/A	N/A	N/A	N/A	32	30	26	28
4	20	N/A	N/A	N/A	N/A	N/A	8	6	20	26
8	14	N/A	N/A	N/A	N/A	N/A	14	14	16	16
11	6	N/A	N/A	N/A	N/A	N/A	0	4	2	2
13	20	N/A	N/A	N/A	N/A	N/A	10	16	6	2
18	12	N/A	N/A	N/A	N/A	N/A	10	8	10	8
21	26	N/A	N/A	N/A	N/A	N/A	N/A	24	22	6
22	30	N/A	N/A	N/A	N/A	N/A	22	28	28	0
23	24	N/A	N/A	N/A	N/A	N/A	16	20	12	16
25	12	N/A	N/A	N/A	N/A	N/A	0	2	6	10
26	18	N/A	N/A	N/A	N/A	N/A	18	18	20	18
27	26	N/A	N/A	N/A	N/A	N/A	22	8	8	12
28	16	N/A	N/A	N/A	N/A	N/A	22	8	6	12
29	16	N/A	N/A	N/A	N/A	N/A	4	4	4	8
30	28	N/A	N/A	N/A	N/A	N/A	26	24	26	20
33	12	N/A	N/A	N/A	N/A	N/A	12	12	10	10
37	28	N/A	N/A	N/A	N/A	N/A	18	22	22	20
38	32	N/A	N/A	N/A	N/A	N/A	28	20	28	28
41	6	N/A	N/A	N/A	N/A	N/A	8	10	12	10
47	18	N/A	N/A	N/A	N/A	N/A	16	20	24	18

Velocity storage attenuation

ID	Intake	Day1	Day2	Day3	Day4	Day5	2wk	1mo	3mo	6mo
2	27	N/A	N/A	N/A	N/A	N/A	8	8	N/A	16
3	26	N/A	N/A	N/A	N/A	N/A	22	18	22	20
6	20	N/A	N/A	N/A	N/A	N/A	6	0	6	0
7	26	N/A	N/A	N/A	N/A	N/A	20	26	10	24
9	22	N/A	N/A	N/A	N/A	N/A	26	22	14	22
10	32	N/A	N/A	N/A	N/A	N/A	28	28	26	22
12	14	N/A	N/A	N/A	N/A	N/A	4	4	6	8
14	30	N/A	N/A	N/A	N/A	N/A	24	6	N/A	0
15	22	N/A	N/A	N/A	N/A	N/A	28	28	N/A	26
16	8	N/A	N/A	N/A	N/A	N/A	4	2	2	2
17	34	N/A	N/A	N/A	N/A	N/A	12	22	12	14
19	24	N/A	N/A	N/A	N/A	N/A	24	18	18	12
20	22	N/A	N/A	N/A	N/A	N/A	18	14	18	12
24	30	N/A	N/A	N/A	N/A	N/A	16	10	10	20
31	14	N/A	N/A	N/A	N/A	N/A	12	10	8	12
32	16	N/A	N/A	N/A	N/A	N/A	18	16	14	12
34	18	N/A	N/A	N/A	N/A	N/A	14	10	18	20
35	36	N/A	N/A	N/A	N/A	N/A	28	32	20	34
36	26	N/A	N/A	N/A	N/A	N/A	20	22	26	30
39	12	N/A	N/A	N/A	N/A	N/A	16	16	14	4
42	12	N/A	N/A	N/A	N/A	N/A	2	2	2	N/A
43	16	N/A	N/A	N/A	N/A	N/A	20	18	19	20
46	30	N/A	N/A	N/A	N/A	N/A	N/A	24	28	28
